# Alkali and Hypochlorite Solution in the Treatment of War Wounds

**Published:** 1918-10

**Authors:** 


***Biological Study in the Action of Alkali and Hypochlorite Solution in the Treatment of War Wounds.*** By Noel Fiessinger and René Clogne, *Revue de Chirurgie*, September-October, 1917.

Any one who has seen wounds treated by débridement followed by Dakin's solution, as advised by Carrel; any one who has seen the dead tissues separate in a few days under this treatment; who has seen the purulent discharge disappear; healthy red granulations appear; and successful secondary sutures made possible between the sixth and fifteenth days, must agree with the favorable reports of Quenu, Pozzi, and Tuffier. Delbet has expressed the view that this solution of Dakin does not act as a sterilizing fluid and the authors, as a result of their work, have reached a similar conclusion. *Hypochlorites in the strength employed have very slight sterilizing power, but as chemical cleansing agents, by proteolysis, they act most energetically.*

Carrel and Ochelly, in their experimental work, showed that the germicidal action of Dakin's solution diminished most markedly in the presence of serum.

Fiessinger and Clogne find that in concentrated albuminous media the germicidal power of Dakin's is almost negligible, as tested *in vitro* on staphyloccus and B. mycoides. In the presence of pus, Dakin's solution in small amounts (proportion of 2 in 10) favors growth of bacteria; in larger amounts (proportion of 6 in 10) it holds growth in check; in very large amounts (proportion of 9 in 10) it destroys bacterial growth. When the infection is introduced into excised tissues and they are treated with Dakin's, provided large amounts of solution are used (much larger than recommended by Carrel), the surface may be sterilized. Rarely can adequate amounts of the solution be employed clinically without an inundation. Clinical experience shows that anaerobes and aerobes persist as long as the wounds contain necrotic tissue, despite regular use of Dakin's solution. Dakin's solution on the other hand undoubtedly hastens the separation of these sloughs and in this way diminishes the bacterial content of wounds.

